# PLGA nanoparticles co-delivering MDR1 and BCL2 siRNA for overcoming resistance of paclitaxel and cisplatin in recurrent or advanced ovarian cancer

**DOI:** 10.1038/s41598-018-25930-7

**Published:** 2018-05-14

**Authors:** Chitra Risnayanti, Yeong-Su Jang, Jinju Lee, Hyung Jun Ahn

**Affiliations:** 1 Center for Theragnosis, Biomedical Research Institute, Korea Institute of Science and Technology, Seoul, 02792 Republic of Korea; 2 Division of Bio-Medical Science &Technology, KIST School, Korea University of Science and Technology, Seoul, 02792 Republic of Korea

## Abstract

The inherent or acquired resistance to paclitaxel and cisplatin, which are commonly used chemotherapeutic agents for ovarian cancer treatment, remains an important issue in chemotherapy of multidrug resistant ovarian cancer. Currently, it is still challenging to deal with the recurrent or advanced stage ovarian cancer. When drug efflux and anti-apoptotic pathways are highly interdependent and also involved in developing the resistance of multidrug resistant ovarian cancer, simultaneous inhibition of both pathways represents the potential targets to enhance the efficacy of chemotherapy. Here, we introduce PLGA nanoparticles system as a “dual RNAi delivery system” to contain both MDR1 and BCL2 siRNA, which is designed for simultaneous inhibition of drug efflux and cell death defense pathways. In the present studies, siRNA-loaded PLGA nanoparticles efficiently elicit the simultaneous suppression of both genes, which consequently shows more enhanced drug-sensitivity than sole suppression of drug efflux or anti-apoptosis in the resistant ovarian cancer cells, owing to the interdependence of both pathways. Our siRNA-loaded PLGA nanoparticles for co-delivering MDR1 and BCL2 siRNA provide an efficient combination therapy strategy to overcome the chemoresistance of paclitaxel and cisplatin on the paclitaxel-resistant SKOV3-TR and cisplatin-resistant A2780-CP20 ovarian cancer respectively.

## Introduction

Multidrug resistance (MDR) that reduces the cytotoxic effects of chemotherapeutics is common and most likely to associate with a relapse of cancer and a failure in the chemotherapy. In an example of advanced ovarian cancer, the diminishing response rate of chemotherapeutics, which is caused by induction of chemoresistance, leads to a 5-year survival of only 30% in patients^1^. Currently, paclitaxel and cisplatin are widely adopted as a standard first-line chemotherapy for ovarian cancer, but the development of such a resistance has limited their use in the recurrent ovarian cancer.

To circumvent such the chemoresistance, functional inhibitors such as cyclosporine A and verapamil have been developed until recently, but most of them did not lead to a clinical approval owing to their undesirable toxicity in body^2,3^. Recently, a number of target genes associated with the chemoresistance in the MDR ovarian cancer cells have been identified, but it is still no easy matter to regulate the malfunction of such genes by using small compounds or monoclonal antibodies^4^.

Over the past few years, considerable progress has been made in our understanding of the mechanisms that underlies drug resistance, and thus recent studies demonstrate that pump and non-pump type of genes are highly associated with the development of chemoresistance on most MDR cancers^4^. Pump type of resistance, induced by overexpression of ATP-binding cassette transporter such as P-glycoprotein (P-gp)^5^, is involved in deporting drugs from the cells, while the representative non-pump type is cell death defense pathway that is activated by anti-apoptotic BCL2 proteins^6^.

As an effort to enhance the drug-sensitivity in the ovarian cancer, previous studies have reported that the combination therapy comprising doxorubicin and BCL2 siRNA increased the apoptosis of SKOV3 ovarian cancer cells, although the selected cancer cell lines were drug-sensitive, rather than drug-resistant^7^. However, such “sole RNAi suppressor” strategy is not an efficient strategy to block the development of chemoresistance on MDR ovarian cancer, because multidrug resistance on the MDR cancer is often determined by a variety of resistance mechanisms, in which pump and non-pump types of resistance is highly interdependent^8^.

Poly(DL-lactide-co-glycolide acid) (PLGA) polymer has been widely used as the efficient carriers for various drug molecules such as small compounds and nucleotides, due to its low toxicity, biodegradability, and sustained release capability^9^. Up to date, several PLGA-based formulations have been approved for use in humans by the Food and Drug Administration^10^. After taken up by cells, PLGA nanoparticles were shown to release their gene contents to the cytoplasmic space over extended periods of time^11^. Therefore, PLGA nanoparticles for delivering siRNA oligo have attracted great interests as an alternative delivery system to the commonly used polycationic delivery systems that are unavoidably toxic and/or non-biodegradable^12^.

Herein, we synthesized PLGA nanoparticles (PLGA NPs) as a co-delivery nanocarrier for MDR1 and BCL2 siRNA by using poly-L-lysine (PPL) as a complexing reagent, and demonstrated that siRNA-loaded PLGA nanoparticles (siRNA@PLGA NPs) could efficiently elicit the simultaneous suppression of drug efflux and anti-apoptosis in the relation of mutual dependence on MDR ovarian cancer cells. When compared with the sole RNAi suppression of drug efflux or anti-apoptotic pathways, this dual RNAi suppression system revealed a marked increase in cytotoxicites of paclitaxel and cisplatin on the paclitaxel-resistant SKOV3-TR and cisplatin-resistant A2780-CP20 ovarian cancer cells, respectively. Thus, our studies provide a potential combination therapy strategy to expand the use of paclitaxel and cisplatin to the therapeutic treatment of recurrent or advanced ovarian cancer including SKOV3-TR and A2780-CP20.

## Results

### Synthesis and characteristics of siRNA-loaded PLGA NPs

When a double emulsion solvent evaporation method is applied to synthesis of siRNA-loaded PLGA NPs, siRNA is dissolved in the aqueous phase, whereas PLGA polymer in the oil phase. To achieve higher encapsulation efficiency, cationic poly-L-lysine (PLL), which is an alternative agent to commonly used polyethylenimine (PEI) to avoid the toxicity associated with PEI’s high positive charge density, was employed to make siRNA less hydrophilic by weakening its negative charge. On gel retardation assay, the charge-charge interactions between PLL and siRNA form the high molecular weight of complexed structures at a 1:1 weight ratio (PLL:siRNA, w/w) corresponding to N:P = 1:1 (Supplementary Fig. S1). The siRNA encapsulation efficiency of PLGA NPs containing PLL/(MDR1 siRNA plus BCL2 siRNA) complexes (*i.e*. siRNA@PLGA NPs) was 71.5 ± 2.1%. Noticeably, when siRNA was encapsulated into PLGA NPs without any aid of PLL, the encapsulation efficiency was less than 2%. These results suggest that the positively charged PLL/siRNA complexes, when compared with the naked siRNA oligo, may be more efficient to complex with the negative charged PLGA. siRNA release studies showed a 35% burst release during the first 1 day, followed by sustained release over 10 days, and the total percent of siRNA release was about 50% in 10 days (Supplementary Fig. S2).

**Figure 1:**
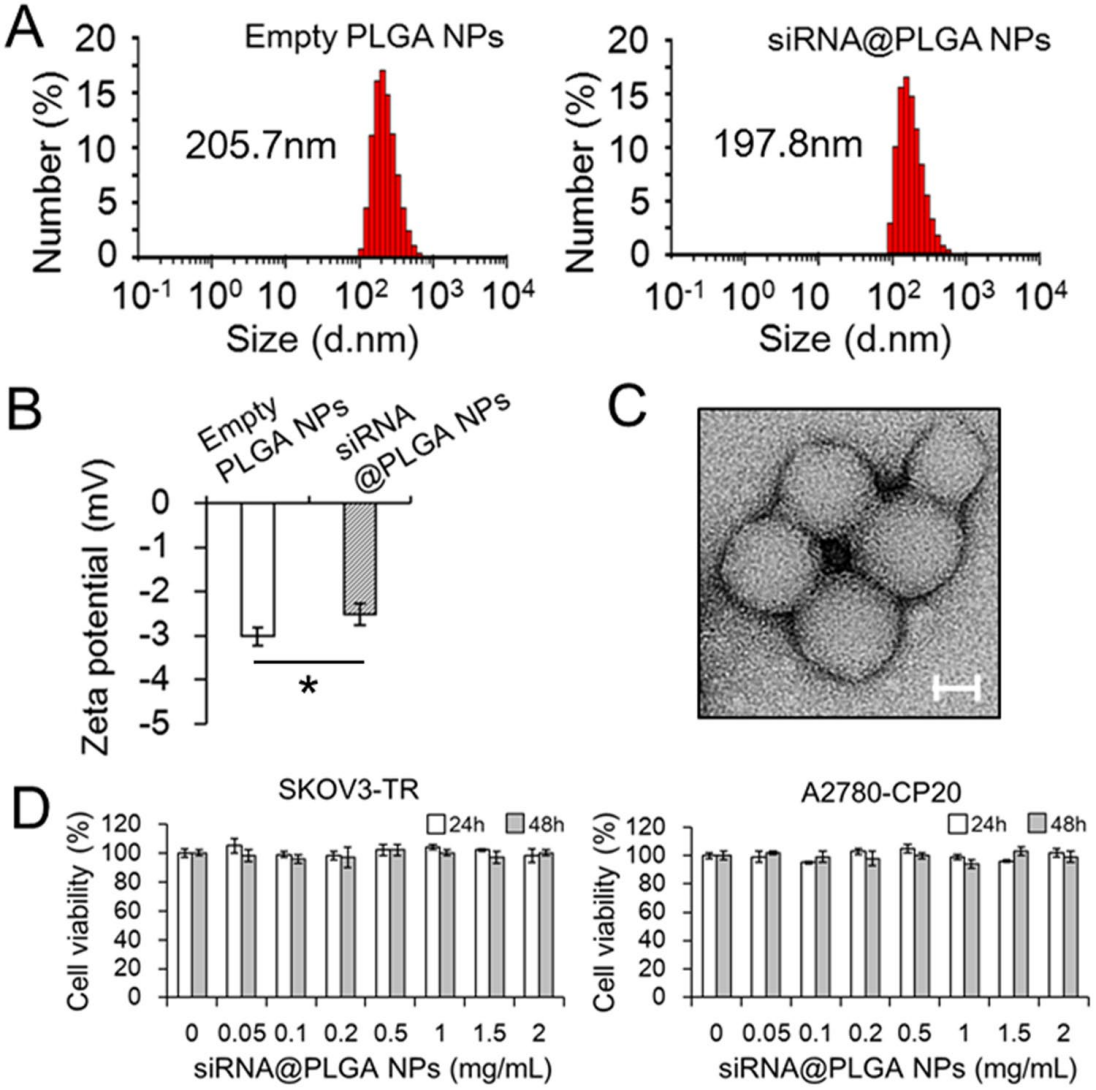
Characterization of siRNA-loaded PLGA nanoparticles. The representative hydrodynamic size distribution profiles (**A**) and zeta potential (**B**) of empty PLGA NPs and PLGA NPs containing MDR1 and BCL2 siRNA (siRNA@PLGA NPs) analyzed on dynamic light scattering (DLS). The negative charge of siRNA@PLGA NPs is slightly lower than that of empty PLGA NPs (*P* < 0.005). The results are presented as the mean ± s.d. (n = 3). (**C**) Transmission electron microscopy images of siRNA@PLGA NPs. Scale bar: 100 nm. (**D**) Cytotoxicity studies of siRNA@PLGA nanoparticles on MDR ovarian cancer cells. Cell viability of SKOV3-TR and A2780-CP20 cancer cells treated with incremental concentrations of siRNA@PLGA NPs was determined by an MTT assay for 24 h and 48 h respectively. The results are presented as the mean ± s.d. from three independent experiments.

The hydrodynamic diameters of empty PLGA NPs and siRNA@PLGA NPs were 205.7 ± 2.5 and 197.8 ± 5.2 nm respectively on dynamic light scattering (DLS) analysis (Fig. 1A). Zeta potential analysis revealed that the negative charge of siRNA@PLGA NPs (−2.51 mV) became slightly lowered than that of empty PLGA NPs (−3.02 mV) due to the positively charged PLL/siRNA complexes (Fig. 1B). TEM image analysis showed that the external surface morphology of siRNA@PLGA NPs was spherical and the averaged diameter was ranging from 180 nm to 200 nm (Fig. 1C).

The cytotoxic effects of siRNA@PLGA NPs on SKOV3-TR and A2780-CP20 human MDR ovarian cancer cells were examined using an MTT assay. Up to extremely high concentrations such as 2 mg/mL, no significant cytotoxic effect was observed until 48 h in either of cell lines (Fig. 1D). These results indicate that PLGA NPs do not cause any severe cytotoxic effect on both MDR cancer cell lines and can be administrated for siRNA delivery in the appropriate concentration ranges to suppress the drug resistance-associated genes without undesirable cytotoxicity. In contrast to cytotoxicity as shown in the use of PEI^13,14^, poly-L-lysine, which was employed as an alternative to PEI, did not induce any cytotoxicity while facilitating a large payload of siRNA. In addition, cytokine release of siRNA@PLGA NPs was examined by using mouse macrophage cells and human lymphoma Ramos cells, because siRNA structures or their carriers may be closely associated with the innate immune responses, which potentially lead to the cytokine release such as TNF-α and INF-α^15^. As a control, release of TNF-α and INF-α was significantly induced in the lipopolysaccharide-treated cells or CpG ODN-treated cells both 4 h and 24 h post-treatment (Supplementary Fig. S3). However, either of empty PLGA NPs or siRNA@PLGA NPs did not induce any appreciable cytokine response. siRNA@lipofectamine complexes, which represent the commercialized siRNA delivery, did not show any remarkable cytokine release, either. Thus, these results indicate that siRNA@PLGA NPs may suppress the expression of target genes in the body without stimulating the innate immune system.

### Cellular uptake of siRNA@PLGA NPs

**Figure 2:**
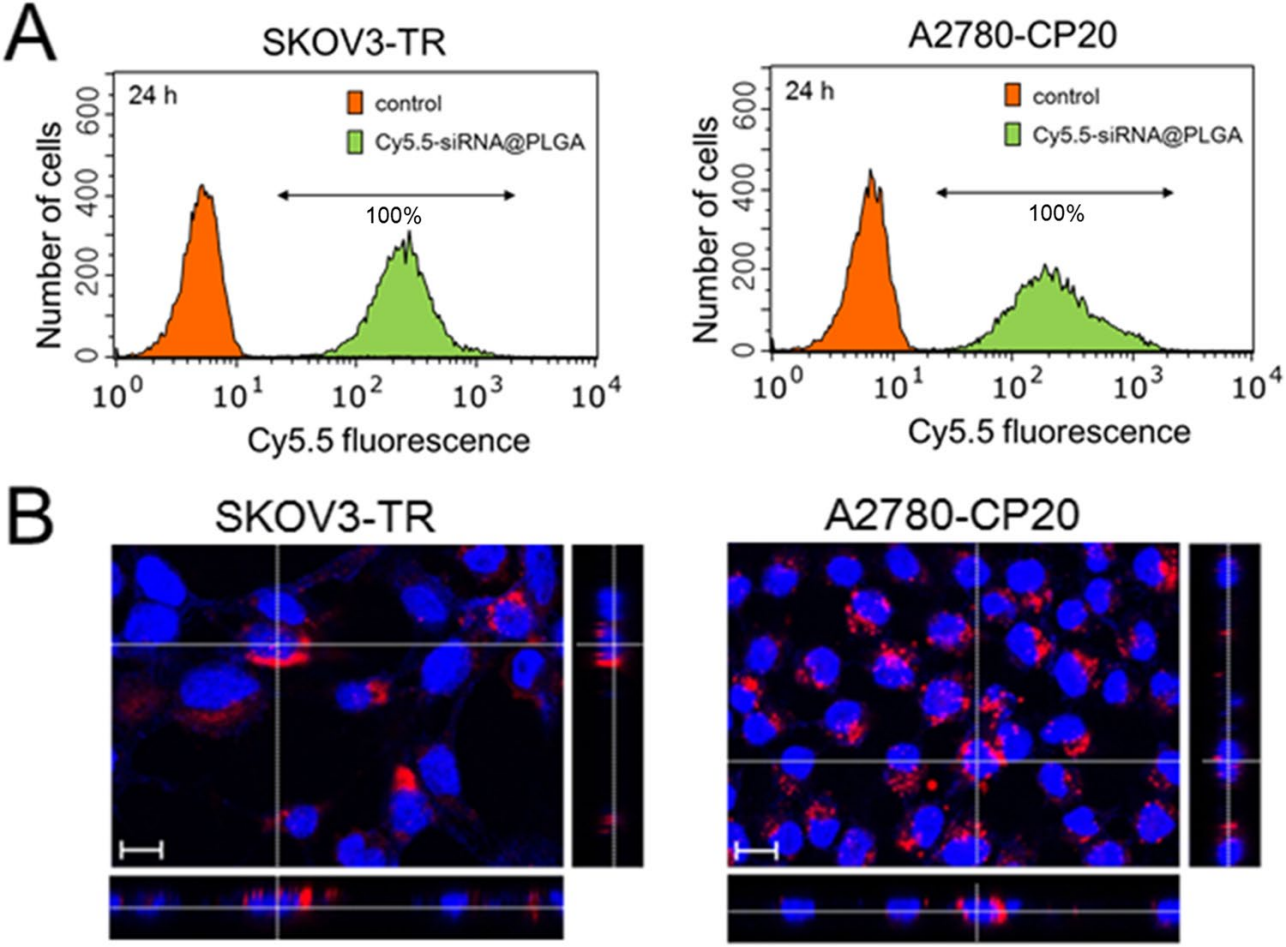
Efficient cellular internalization of siRNA@PLGA NPs on MDR ovarian cancer cells. (**A**) Flow cytometric histogram showing cellular internalization of the fluorescent siRNA@PLGA NPs on SKOV3-TR and A2780-CP20 cancer cells respectively. The resistance cell lines were treated with fluorescent Cy5.5-siRNA@PLGA NPs, and 24 h later, sorted. A prefixed gate region was allowed only for Cy5.5 fluorescence. The untreated cells are indicated as a control. (**B**) Z-stack confocal microscopic images of SKOV3-TR and A2780-CP20 cancer cells treated with Cy5.5-labeled siRNA@PLGA NPs. Cy5.5-siRNA and DAPI dyes are represented by red and blue, respectively. Scale bar: 10 μm.

To assess the cellular uptake of siRNA@PLGA NPs on MDR cancer cell lines, the fluorescent Cy5.5-siRNA-loaded PLGA NPs were treated to MDR SKOV3-TR cells and A2780 cells respectively. On flow cytometry analysis, the increased fluorescence intensities of Cy5.5-siRNA@PLGA NPs-treated cell lines indicated that PLGA NPs efficiently delivered the encapsulated siRNA into both MDR ovarian cancer cell lines (Fig. 2A). On the confocal microscopic images, Cy5.5-siRNA@PLGA NPs-treated cancer cells exhibited the intracellular punctate fluorescence signals in the cytoplasm compartment of both cell lines, whereas there was little or no fluorescence in the nucleus (Fig. 2B). All things considered, these results show that siRNA@PLGA NPs can be substantially internalized into MDR ovarian cancer cells. Particularly, it is worth noting that the cellular uptake of PLGA nanoparticles can be enhanced by the use of poly-L-lysine in such a way that its positive charge can neutralize the negative charge of PLGA nanoparticles^16^.

### Intrinsic overexpression of MDR1 and BCL2 genes on drug-resistant ovarian cancer cell lines

**Figure 3:**
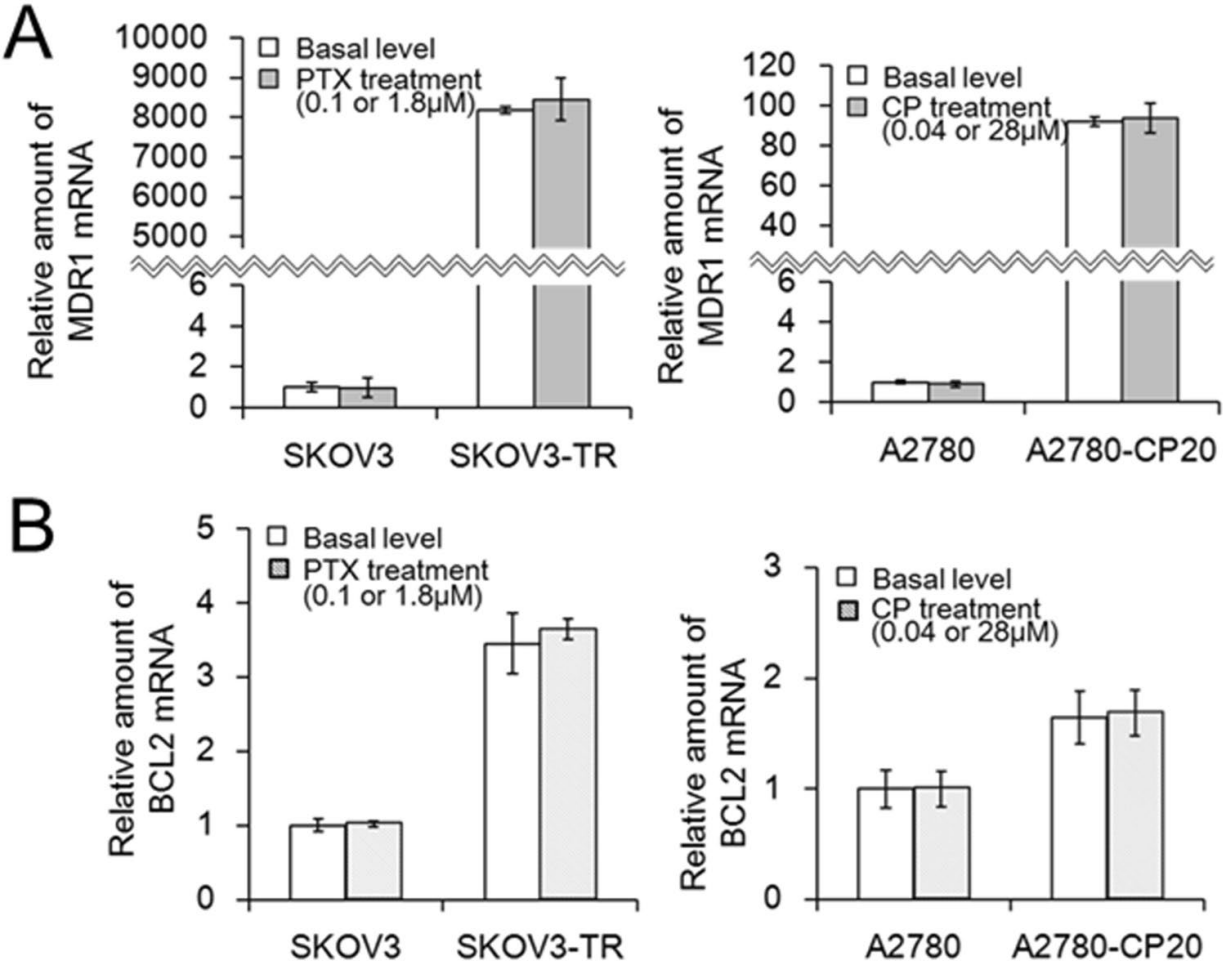
Intrinsic overexpression of MDR1 and BCL2 genes associated with drug-resistance on MDR ovarian cancer cells. The amounts of MDR1 (**A**) and BCL2 mRNA (**B**) on SKOV3 or A2780 parent and resistant cells were measured without or in response to chemotherapeutics treatment. 0.1 μM and 1.8 μM of paclitaxel (PTX) were treated to SKOV3 and SKOV3-TR cells, respectively, and 0.04 μM and 28 μM of cisplatin (CP) were treated to A2780 and A2780-CP20 cells, respectively. qRT-PCR was performed to quantitatively determine the amounts of each mRNA. The results are represented as the mean ± s.d. (n = 3). The differences between intrinsic and induced expression levels in MDR1 or BCL2 genes on both cell lines were estimated as statistically nonsignificant under *p* < 0.05.

Using quantitative mRNA and immunoblotting analysis, the intrinsic overexpression of both MDR1 and BCL2 genes on SKOV3-TR and A2780-CP20 resistant cell lines was compared to those of SKOV3 and A2780 parent cell lines. First, qRT-PCR analysis showed that MDR1 mRNA levels were 8192 times higher on SKOV3-TR cells than those on SKOV3 cells, and 91.8 times higher on A2780 cells than those on A2780-CP20 cells (Fig. 3A). Also, BCL2 mRNA levels were 3.5 times higher on SKOV3-TR cells than those on SKOV3 cells, and 1.6 times higher on A2780 cells than those on A2780-CP20 cells (Fig. 3B). Noticeably, the mRNA levels of MDR1 genes revealed a more distinct difference between parent and resistant cell lines than those of BCL2 genes, as SKOV3-TR cells showed the extremely overexpressed MDR1 levels. When paclitaxel was administered to both cell lines, we did not observe any appreciable increase in the mRNA levels of both genes. Similarly, cisplatin treatment did not lead to any appreciable elevation of mRNA levels of both genes on the A2780 parent and resistant cell lines. These results show that the intrinsically overexpressed status of MDR1 and BCL2 genes may be highly correlated with drug resistance on both resistant cell lines, and also indicate that the resistance associated with MDR1 and BCL2 genes may be intrinsic on both resistant cell lines, rather than acquired. Immunoblotting analysis also confirmed that SKOV3-TR and A2780-CP20 resistant cell lines intrinsically maintained the elevated expression levels on both P-glycoproteins and BCL2 proteins in contrast to their parent cell lines (Supplementary Fig. S4).

### Quantitative measurement of MDR1 and BCL2 gene silencing

**Figure 4:**
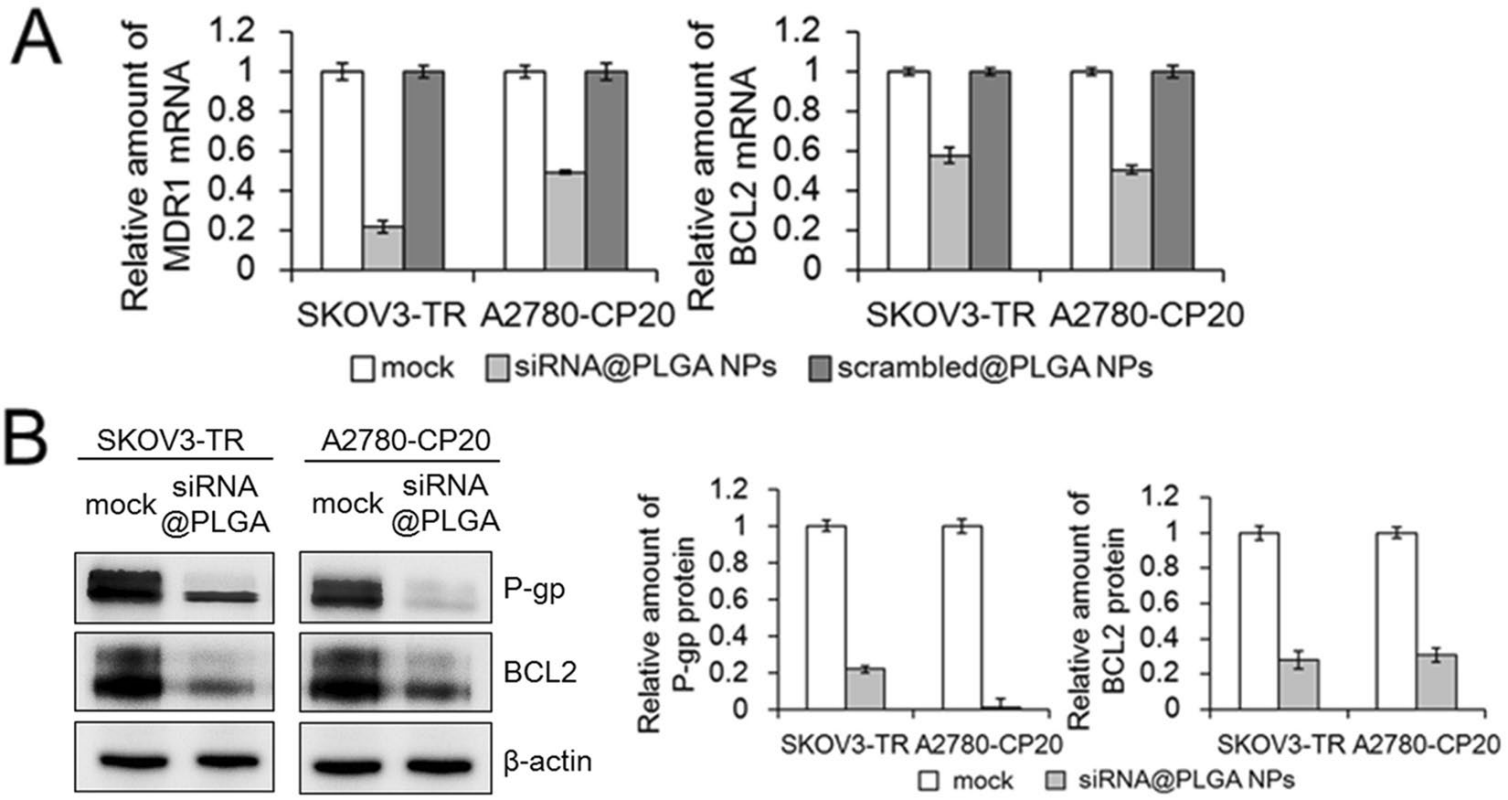
Quantitative measurement of gene silencing for MDR1 and BCL2 on MDR ovarian cancer cells treated with siRNA@PLGA NPs. (**A**) The mRNA levels of MDR1 and BCL2 were determined by qRT-PCR method 24 h after SKOV3-TR and A2780-CP20 cells were treated with PLGA NPs containing MDR1 and BCL2 siRNA. (**B**) The expression levels of P-glycoproteins and BCL2 proteins were measured by immunoblotting method 24 h after SKOV3-TR and A2780-CP20 cells were treated with siRNA@PLGA NPs. In the figure are reported the cropped gels/blots obtained by each protein evaluation. All gels were run in the same experimental conditions and a representative immunoblotting image was presented. The levels of P-glycoproteins or BCL2 proteins were quantitatively plotted relative to those of mock sample cells. All results are represented as the mean ± s.d. (n = 3).

To examine the gene silencing effects of siRNA@PLGA NPs, the reduction of MDR1 and BCL2 mRNA was quantitatively measured by qRT-PCR method on SKOV3-TR and A2780-CP20 ovarian cancer cells respectively. The levels of MDR1 and BCL2 mRNA in the siRNA@PLGA NPs-treated SKOV3-TR cells were noticeably decreased by ~78% and ~42% respectively, compared to those in the untreated SKOV3-TR cells (Fig. 4A). Scrambled siRNA@PLGA NPs as a negative control could not down-regulate either MDR1 mRNA or BCL2 mRNA. The levels of MDR1 and BCL2 mRNA were also decreased in the siRNA@PLGA NPs-treated A2780-CP20 cells by ~51% and ~50% respectively, but not in the scRNA@PLGA NPs-treated A2780-CP20 cells. On the other hand, siRNA@PLGA NPs containing the alternative siRNA sequences for MDR1 or BCL2 mRNA showed the similar levels of mRNA decrease both in the siRNA@PLGA NPs-treated SKOV3-TR and A2780-CP20 cells (data not shown), which indicates that siRNA-mediated suppression of MDR1 and BCL2 genes is highly sequence-specific and is not derived from the off-target effect of chosen siRNA.

To more examine the silencing effects of siRNA@PLGA NPs, we carried out the western blotting analysis using the primary antibodies specific for P-glycoproteins and BCL2 proteins. The levels of P-glycoproteins and BCL2 proteins in the siRNA@PLGA NPs-treated SKOV3-TR cells were significantly reduced by ~78% and ~72% respectively (Fig. 4B). In addition, the levels of P-glycoproteins and BCL2 proteins in the siRNA@PLGA NPs-treated A2780-CP20 cells were remarkably decreased by ~94% and ~69%. Taken together with these results, such significant decrease in each of mRNA and protein levels for MDR1 and BCL2 indicates that siRNA@PLGA NPs are able to efficiently down-regulate expression of both genes highly associated with pump and non-pump type of resistance.

### Suppression of drug efflux on siRNA@PLGA NPs-pretreated MDR cells

**Figure 5:**
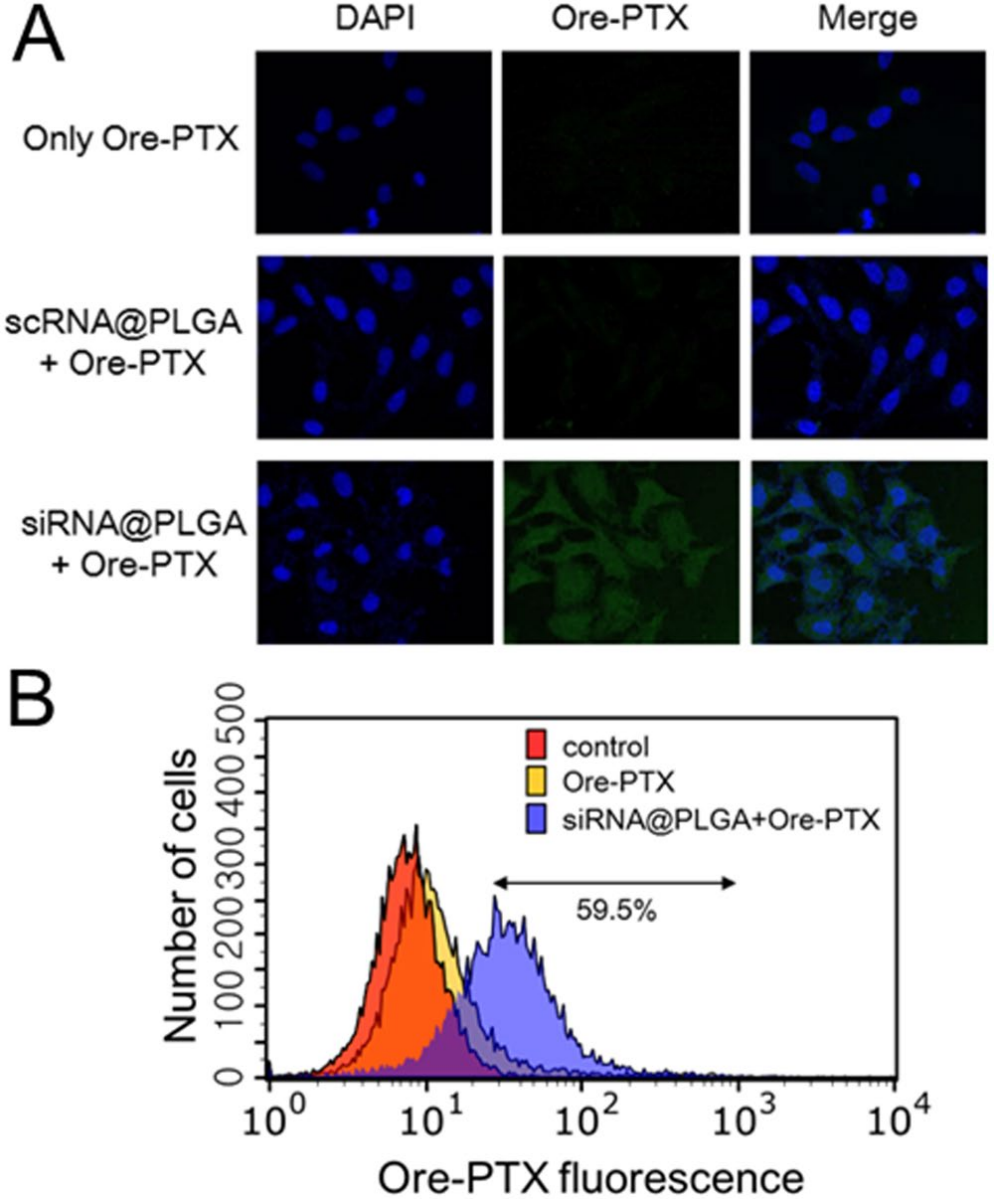
Increased intracellular concentration of drugs on siRNA@PLGA NPs-pretreated MDR cancer cells. (**A**) Confocal microscopic images demonstrating the suppression of drug efflux on MDR SKOV3-TR cells. Cells were first pretreated with siRNA@PLGA NPs, and 24 h later, administrated with 0.1 μM fluorescent paclitaxel conjugates (Oregon green-488 paclitaxel, Ore-PTX) for monitoring their intracellular localization. Cellular confocal microscopic evaluation was carried out 12 h after drug treatment. As a control, suppression of drug efflux by the scrambled siRNA@PLGA NPs was also compared. (**B**) Flow cytometry analysis for measuring the intracellular accumulation of fluorescent paclitaxel conjugates on SKOV3-TR cells. A prefixed gate region was allowed only for Oregon green-488 fluorescence. A representative histogram among three independent experiments was shown here.

Because P-glycoproteins were expressed at levels sufficient to confer the overactivated drug efflux in the MDR ovarian cancer cells as shown above, accumulation of Oregon green-488 paclitaxel (Ore-PTX), which is a fluorescent paclitaxel conjugate as well as a fluorescent substrate of P-glycoproteins, was assessed on the siRNA@PLGA NPs-pretreated SKOV3-TR cells using confocal microscopic images. First, any fluorescent signals were not observed on the free Ore-PTX-treated SKOV3-TR cells due to the overactivity of drug efflux (Fig. 5A). However, siRNA@PLGA NPs-pretreated SKOV3-TR cells exposed the significantly accumulated Ore-PTX drugs within the cells, which indicate that RNAi-mediated inhibition of drug efflux lead to the efficient accumulation of drugs. The scrambled siRNA-loaded PLGA NPs did not show any appreciable fluorescent signal due to the lack of suppression of P-glycoprotein function. When the intracellular localization of Alexa Fluor 546-cisplatin (Alexa-CP) was monitored on A2780-CP20 cells, highly accumulated Alexa-CP drugs were observed within the siRNA@PLGA NPs-pretreated cells, but little or no fluorescent drug was seen both within the scRNA@PLGA NPs-pretreated cells and free Alexa-CP-treated cells (Supplementary Fig. S5A).

In addition, the inhibition of cellular efflux of drugs on the SKOV3-TR cell lines was examined using flow cytometry analysis and fluorescent Ore-PTX drugs. With respect to the untreated SKOV3-TR cells, only 7.6 ± 0.2% of cells showed the intracellular accumulation of drugs (Fig. 5B). However, the intracellular accumulation significantly increased by 59.5 ± 4.8% in the siRNA@PLGA NPs-pretreated SKOV3-TR cells. The scrambled siRNA@PLGA NPs-pretreated SKOV3-TR cells showed little or no intracellular accumulation of drugs as expected (data not shown). Similarly, flow cytometry analysis showed 64.7% of intracellular accumulation of Alexa-CP20 drugs within the siRNA@PLGA NPs-pretreated A2780-CP20 cells with respect to the untreated cells or scRNA@PLGA NPs-treated cells (Supplementary Fig. S5B). These results show that siRNA@PLGA NPs can efficiently suppress the function of P-glycoproteins in the MDR ovarian cancer cells and thus increase the accumulation of intracellular drugs, which is highly associated with potential tumor responsiveness to chemotherapeutics.

### Induction of cell death on MDR ovarian cancer cells by combination treatment of chemotherapeutics and siRNA@PLGA NPs

**Figure 6:**
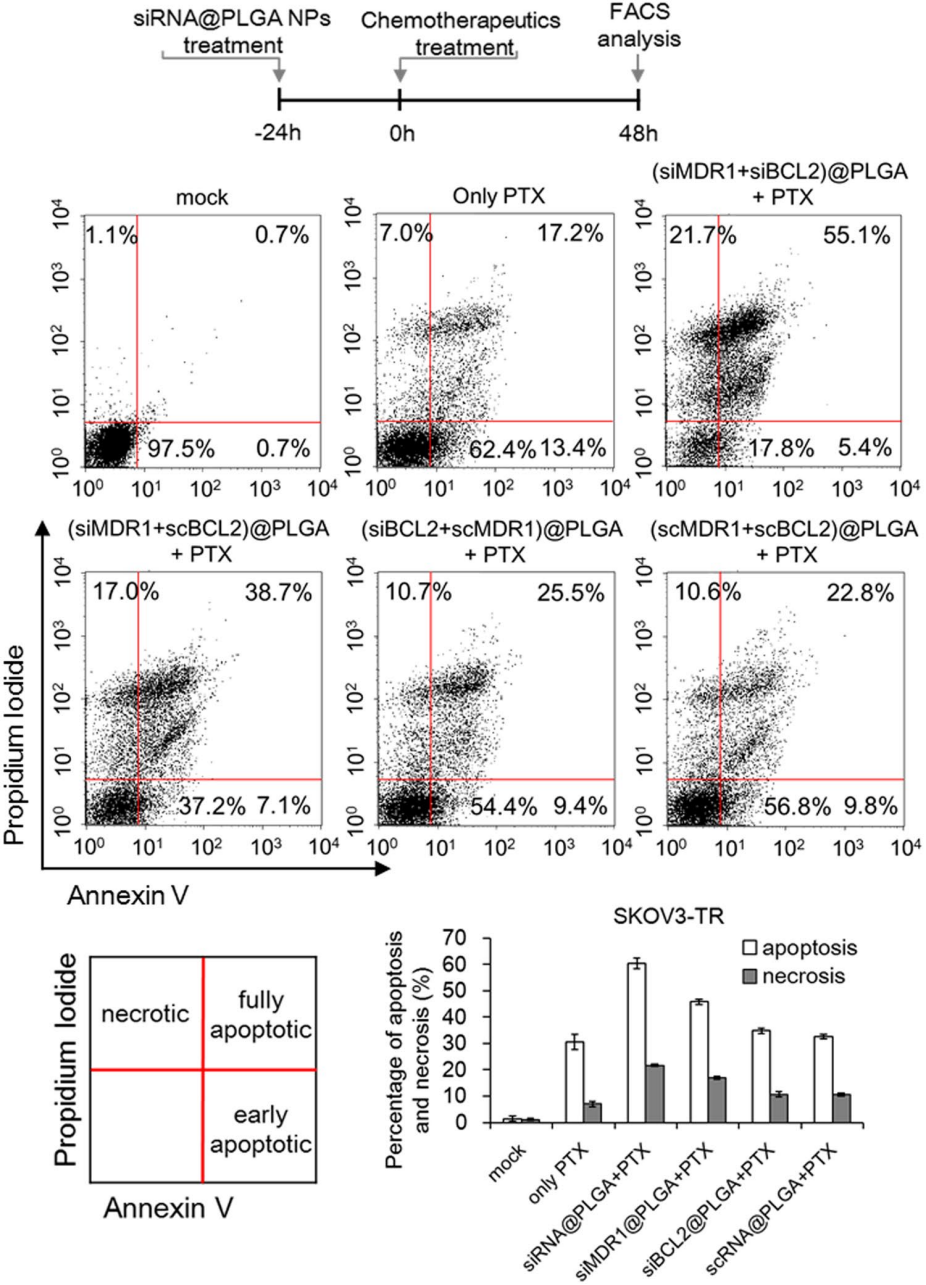
Induction of apoptotic and necrotic cell death on MDR ovarian cancer cells by combination treatment. MDR SKOV3-TR cells were treated sequentially with PLGA NPs containing MDR1 and BCL2 siRNA (37.5 nM, respectively) and paclitaxel (5 μM), as represented in a schematic diagram. Based on flow cytometry, induction of apoptosis and necrosis was investigated in triplicate, and a representative experiment set is demonstrated here. Each percentage of fully apoptotic, early apoptotic, and necrotic cells were indicated in the corresponding quadrant, and also plotted below. Both the fully and early apoptotic cells are included in the percentage of apoptosis, and the results are shown as the mean ± s.d. (n = 3).

Cell death induced by chemotherapeutics 24 h after siRNA@PLGA NPs treatment was investigated on MDR SKOV3-TR and A2780-CP20 cancer cells by using propidium iodine and Annexin V double staining method. First, free paclitaxel, as a control, induced cell death (apoptosis plus necrosis) up to 37.6% on SKOV3-TR cells (Fig. 6). Paclitaxel combined with (siMDR1 + scBCL2)@PLGA NPs or (siBCL2 + scMDR1)@PLGA NPs as a sole siRNA suppressor system could induce cell death up to 62.8% or 45.6%, respectively. However, paclitaxel combined with siRNA@PLGA NPs as a dual siRNA suppressor system was able to induce more cellular apoptosis and necrosis up to 82.2%. Paclitaxel combined with scrambled siRNA@PLGA NPs did not lead to any notable increase in cell death, compared to free paclitaxel. Also, the scrambled siRNA@PLGA NPs alone did not induce any detectable cell death as expected (data not shown).

Next, free cisplatin, as a control, induced cell death up to 38.2% on A2780-CP20 cells, while cisplatin combined with (siMDR1 + scBCL2)@PLGA NPs or (siBCL2 + scMDR1)@PLGA NPs could induce cell death up to 52.0% or 43.1%, respectively (Supplementary Fig. S6). However, cisplatin combined with siRNA@PLGA NPs could increase cell death up to 94.5%. As expected, the scrambled siRNA@PLGA NPs did not affect the drug-sensitivity of cisplatin on A2780-CP20 cells. Taken together, these data clearly show that siRNA@PLGA NPs can efficiently overcome the drug-resistance in the MDR SKOV3-TR and A2780-CP20 cells, and also indicate the simultaneous inhibition of both genes is more efficient to enhance the cytotoxicity of chemotherapeutics on the resistant ovarian cancer cells, compared with the single inhibition of either MDR1 or BCL2 genes.

### Enhanced cytotoxicity of chemotherapeutics combined with siRNA@PLGA NPs

**Figure 7:**
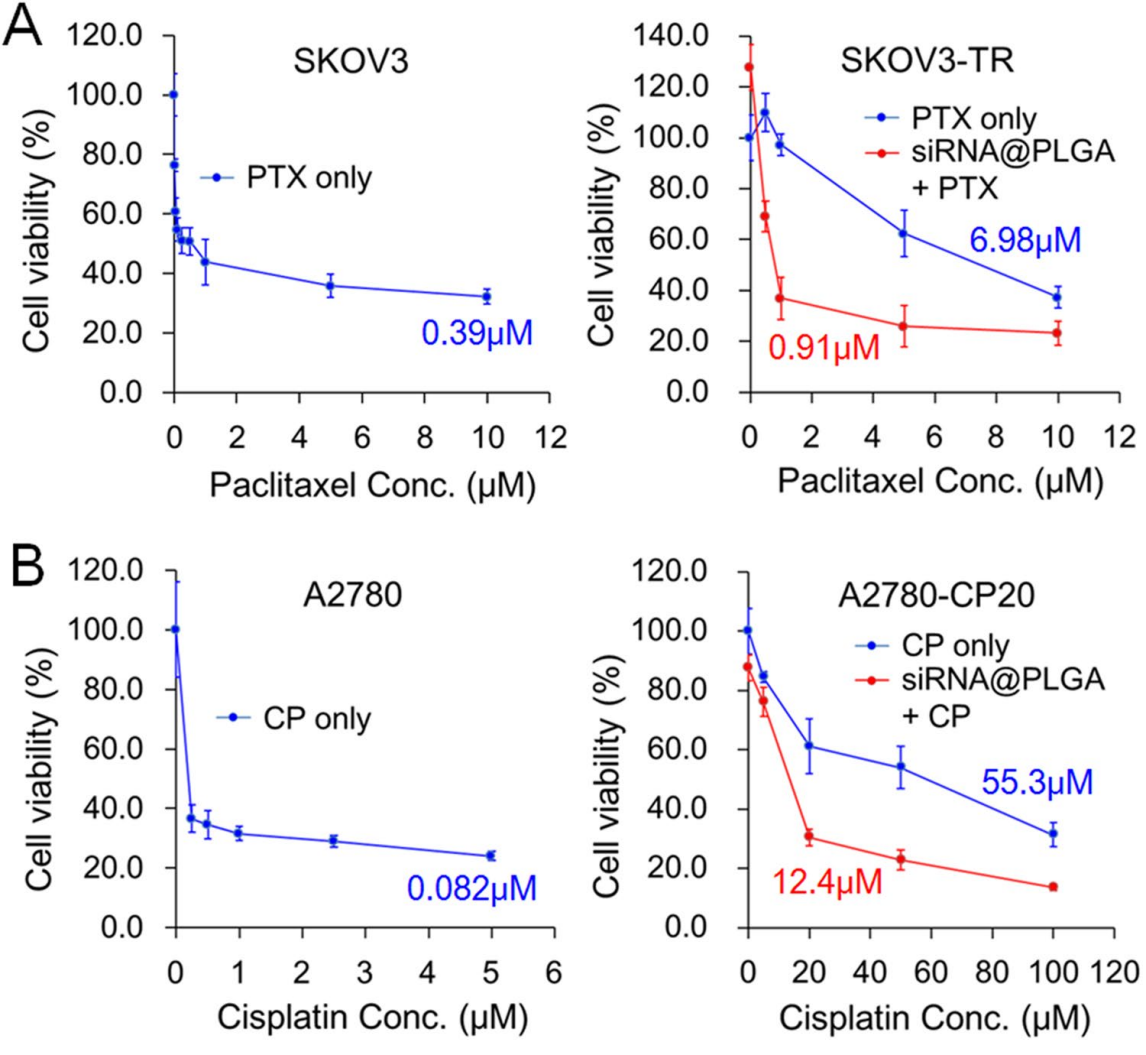
Increased drug-sensitivity of chemotherapeutics on MDR ovarian cancer cells in the presence of siRNA@PLGA NPs. Suppression of cell growth of SKOV3 parent and resistant cell lines (**A**) or A2780 parent and resistant cell lines (**B**) under incremental concentrations of paclitaxel (PTX) or cisplatin (CP) in the absence or in the presence of siRNA@PLGA NPs. MDR cancer cells were pretreated with siRNA@PLGA NPs (0.28 mg/mL) containing MDR1 and BCL2 siRNA (37.5 nM respectively), and 24 h later, treated with paclitaxel or cisplatin for 48 h. Cell viability was assessed by an MTT assay. IC_50_ values of chemotherapeutics were estimated by using nonlinear regression analysis of Origin 8 data analysis software. Each IC_50_ value is presented beside the corresponding plot. Data were represented as mean ± s.d. from three independent experiments. Any marked promotion of drug-sensitivity on both SKOV3 and A2780 parent cell lines was not seen and a higher dosage of siRNA@PLGA NPs, ranging from 0.4 mg/mL to 0.9 mg/mL, did not yield any notable difference in IC_50_ (data not shown).

When cell viability of drug-sensitive or drug-resistant ovarian cancer cell lines was compared under incremental concentrations of chemotherapeutics by using MTT assay, IC_50_ value of paclitaxel on paclitaxel-resistance SKOV3-TR (6.98 μM) was approximately 18.0 times higher than that on SKOV3 parent cell line (0.39 μM) (Fig. 7A and Supplementary Table S2). Given that IC_50_ value reflects the cellular drug-sensitivity to chemotherapeutics, such a remarkable difference in IC_50_ value indicates that SKOV3-TR cell line has significantly higher drug-resistance to paclitaxel than SKOV parent cell line. However, the combined treatment of paclitaxel with siRNA@PLGA NPs lead to a significantly decreased IC_50_ value (0.91 μM), which is 7.65 times less than that of free paclitaxel in the absence of siRNA@PLGA NPs.

Next, cisplatin was highly effective in inducing cell death on A2780 parent cell line with IC_50_ value of 0.082 μM, whereas A2780-CP20 cells showed a high drug-resistance against cisplatin, of which IC_50_ value was 674.4 times greater (55.3 μM) than that on parent cell line (Fig. 7B). On the siRNA@PLGA NPs-pretreated A2780-CP20 cells, the IC_50_ value of cisplatin (12.4 μM) was 4.46 times less than that of free cisplatin (55.3 μM). Taken together, these data show the simultaneous blocking of MDR1 and BCL2 genes, which are mediated by siRNA@PLGA NPs, enables to efficiently promote the sensitivity of drugs on MDR ovarian cancer cell lines.

## Discussion

In the current studies, we demonstrate that PLGA nanoparticles containing both MDR1 and BCL2 siRNA can efficiently suppress the function of both genes on the MDR ovarian cancer cells. When combined with pretreatment of siRNA@PLGA NPs, the IC_50_ value of paclitaxel and cisplatin, which reflects tumor responsiveness to drugs, significantly decreased by 7.65 and 4.46 times on SOV3-TR and A2780-CP20 cells respectively, finally promoting their therapeutic effects on the MDR ovarian cancer cells. Up to date, the inherent or acquired resistance to paclitaxel and cisplatin, although they are commonly used chemotherapeutics for ovarian cancer, has limited their use in the treatment of MDR ovarian cancer patients. Thus, our studies suggest siRNA@PLGA NPs as the potential therapeutic tool to overcome the chemoresistance of paclitaxel and cisplatin on the SOV3-TR and A2780-CP20 cells.

The previous combination therapies exploiting the “sole RNAi suppressor system” against drug efflux gene in the MDR cancer have revealed the needs to block multiple gene pathways that are highly interdependent, because a robust accumulation of drugs within the cells, caused by suppression of pump phenotype, may proportionally induce the significant activation of anti-apoptotic machinery for cell death defense, thus resulting in an unfavorable therapeutic effects^17^. Our siRNA@PLGA nanoparticles show that a dual RNAi suppressor system is more efficient to overcome the drug resistance in the MDR ovarian cancer cells than a sole RNAi suppressor system, especially when multiple genes in the relation of interdependence are involved in the drug resistance.

PLGA nanoparticles have provided the efficient delivery systems for chemotherapeutics due to their low cytotoxicity, which is the critical parameter for medical applications^18^. However, polycationic reagents such as polyethylenimines, which are requisitely employed for a large payload of siRNA within PLGA NPs, has limited the use of PLGA NPs in the development of RNAi-based therapeutics due to their potential toxicity^13,14^. The current studies show that poly-L-lysine, an alternative reagent to avoid the toxicity associated with PEI, does not induce any cellular cytotoxicity, while facilitating a large payload of siRNA in the formulation of PLGA NPs. Several previous studies have shown that PLGA nanoparticles are non-toxic and biocompatible in contrast to other carriers such as chitosan and DOPC^19^, and particularly, suitable for *in vivo* siRNA delivery due to the enhanced permeability and retention (EPR) effect^20^. Following endocytosis for cellular internalization, PLGA nanoparticles can escape the endo-lysosomal compartments and slowly release the payload of siRNA into the cytoplasm^11^, as shown in the Supplementary Fig. S2. Thus, these advantages indicate that PLGA nanoparticles have great potential for siRNA-mediated therapeutic applications as well.

To maximize the therapeutic effects of drugs in the combination therapy with siRNA, their delivery system requires the precisely controlled time intervals between gene silencing and drug unloading, in the way that suppression of resistance phenotypes is completed and subsequently drugs are unloaded. In the current studies, siRNA@PLGA NPs are administered 1 day earlier than drugs for the optimal suppression of chemoresistance, whereas simultaneous administration of drugs and siRNA@PLGA NPs showed little or no suppression of chemoresistance (data not shown). However, a better understanding of the controlled release of drugs and siRNA *in vivo* is still required for the clinical applications.

Herein, our siRNA@PLGA nanoparticles, as a dual RNAi suppressor system on the MDR ovarian cancer cells, provide an efficient combination therapy strategy to overcome the chomoresistance of paclitaxel and cisplatin on MDR SKOV3-TR and A2780-CP20 ovarian cancer, as well as to expand the use of traditional chemotherapeutics to the treatment of recurrent or advanced ovarian cancer.

## Methods

### Materials

Chemically synthesized siRNA against MDR1 and BCL2 gene and scrambled siRNA were obtained from Bioneer Co. (Daejon, Korea) with the sequences shown in Supplementary Table S1. Polyvinyl alcohol (PVA, 80% hydrolyzed, M_w_ 9–10 kDa), poly(D,L-lactide-co-glycolide) (M_w_ 10–12 kDa), poly-L-lysine (PLL), thiazolyl blue tetrazolium bromide, cisplatin, and paclitaxel were purchased from Sigma-Aldrich (UK), and chloroform was from Biosesang (Korea). Fluorescent paclitaxel conjugates (Oregon green-488 paclitaxel) was purchased from Invitrogen, and fluorescent cisplatin conjugates (Alexa Fluor 546-cisplatin) from Molecular Probes (Eugene, US). Antibodies specific for BCL2 and MDR1 were purchased from Cell Signaling (US), and HRP-conjugated anti-rabbit IgG from Abcam (US). The MDR SKOV3-TR and MDR A2780-CP20 cancer cells were obtained from American Type Culture Collection (ATCC, US).

### Preparation of siRNA-loaded PLGA NPs

PLGA NPs containing siRNA against MDR1 and BCL2 genes were synthesized by using the double emulsion solvent evaporation (w_1_/o/w_2_) method as previously reported^21^. Briefly, Each 50 μg of MDR1 and BCL2 siRNA were mixed with 100 μg of PLL in 200 μL of nuclease-free water (N/P = 1), and the resulting solution was further mixed with 2 mL of chloroform containing 20 mg of PLGA. The mixture was emulsified by using a sonicator equipped with a microtip probe (Branson ultrasonic processor, Thermo Fisher Scientific, US) at 4 °C for 1 m to produce a primary w_1_/o emulsion. To stabilize PLGA NPs and reduce their surface tension, 10 mL of 1% PVA in deionized water were poured into the primary emulsion solution, which is further emulsified by a microtip probe sonicator at 4 °C for 5 m to form a w_1_/o/w_2_ double emulsion. The resulting emulsion was stirred to completely evaporate chloroform at room temperature. After the suspension of siRNA@PLGA NPs was washed with deionized water, siRNA@PLGA NPs were collected by using a centrifuge at 15,700 × g for 50 m at 4 °C. This washing process was repeated three times, and then siRNA@PLGA NPs were freeze-dried.

The siRNA encapsulation efficiency was calculated according to the following procedures. First, siRNA@PLGA NPs were dissolved in PBS buffer, and then centrifuged at 15,700 × g for 50 m at 4 °C. The concentration of siRNA in the supernatant was determined using Nanodrop spectrophotometer (Thermo Fisher Scientific, US) to calculate the amount of un-encapsulated siRNA, and consequently, the amount of encapsulated siRNA was calculated by extracting the amount of un-encapsulated siRNA from total amount of siRNA loaded. The siRNA encapsulation efficiency was measured using the following Eqs.:

$${\rm{siRNA}}\,{\rm{encapsulation}}\,{\rm{efficiency}}\,( \% )=({\rm{amount}}\,{\rm{of}}\,{\rm{encapsulated}}\,\mathrm{siRNA}/\mathrm{amount}\,{\rm{of}}\,{\rm{total}}\,{\rm{siRNA}}\,{\rm{loaded}})\times 100 \% $$

The weight ratio of siRNA and PLL was determined by using gel retardation assay. Briefly, 1 μg of siRNA in 10 μL of nuclease-free water was mixed with various amounts of PLL ranging from 0.25 μg to 1 μg, and then the mixture were subjected to 2% agarose gel electrophoresis. The bands on the gel were stained with SYBR Gold.

### Characterization of siRNA-loaded PLGA NPs

The hydrodynamic diameter and zeta potential of siRNA@PLGA NPs were measured using dynamic light scattering. Freeze-dried siRNA-loaded PLGA NPs were re-dispersed in deionized water (0.1 mg/mL) by gentle sonication for 1 m, and then filtered using syringe filter (0.22 μm). The measurement was carried out on the undiluted samples using Zetasizer Nano ZS (Malvern Instrument, US), which was equipped with a 633 nm of laser at a scattering angle of 173°. The morphology and particle size of siRNA@PLGA NPs was measured using transmission electron microscopy (TEM) images as described previously^22^. On TEM analysis of polymeric nanoparticles, CM30 electron microscope (Philips, US) operated at 80 kV acceleration voltage and a droplet of 2.5 wt% aqueous uranyl acetate was used for negative staining.

### Release studies of siRNA

Using Cy5.5-labeled siRNA@PLGA NPs, release kinetics of siRNA from PLGA nanoparticles was examined. Cy5.5-siRNA@PLGA NPs were suspended in PBS buffer with gentle stirring, and then a small volume of sample solutions were taken in triplicate at the indicated time points. After the nanoparticles were centrifuged and disintegrated in DMSO, the fluorescence intensity of Cy5.5-siRNA was detected by Xenogen IVIS Lumina imaging system (Perkin Elmer).

### Cell culture and transfection

Each of resistant SKOV3-TR and A2780-CP20 human ovarian cancer cells were seeded in 96-well plates (1 × 10^4^ cells/well) in RPMI 1640 medium supplemented with 10% FBS in a 37 °C and 5% CO_2_ incubator, and cultured overnight to reach 70% confluency. The medium were replaced with FBS-free RPMI 1640, and subsequently siRNA@PLGA NPs solution was treated to each well for 3 h (the final concentration of siRNA@PLGA NPs was 0.28 mg/mL, where each concentration of loaded MDR1 and BCL2 siRNA was 37.5 nM). Next, the medium were replaced with FBS-containing RPMI 1640, and the cells were further cultured for the indicated period.

### Cytotoxicity studies of siRNA@PLGA NPs

*In vitro* cytotoxicity of siRNA@PLGA NPs was examined on SKOV3-TR and A2780-CP20 cancer cells respectively by an MTT assay method following the protocol described elsewhere^23^. Briefly, SKOV3-TR cells plated on 96-well plates were cultured overnight to 70% confluency as aforementioned. Next, the cells were transfected with 0.02–2.0 mg/mL of siRNA@PLGA NPs per well, and further incubated for 24 h. After 3-(4,5-Dimethylthiazol-2-yl)-2,5-Diphenyltetrazolium Bromide was supplemented to each well, the cells were incubated for 4 h. The quantity of produced formazan crystals were subsequently analyzed using a Spectra MAX340 microplate reader (Molecular Device, US). The cytotoxicity studies of siRNA@PLGA NPs on A2780-CP20 cancer cells were carried out in the same way.

### Measurement of gene silencing using qRT-PCR analysis and western blotting

The amounts of MDR1 and BCL2 mRNA on the siRNA@PLGA NPs-treated SKOV3-TR cells were estimated by qRT-PCR (quantitative reverse transcription PCR) method. First, MDR SKOV3-TR cancer cells were plated on 96-well plates, and further cultured overnight to reach 70% confluency. Subsequently, the cells were transfected with siRNA@PLGA NPs (0.28 mg/mL) containing 37.5 nM siRNA for each of MDR1 and BCL2. At 3 h post-transfection, the cells were washed twice with DPBS buffer and further incubated in RPMI 1640 medium containing 10% FBS for 21 h. For measurement of mRNA, total RNA from cells could be isolated by using RNeasy Mini Kit (Qiagen) and its cDNA was enzymatically synthesized by using High Capacity cDNA Archive Kit (Thermo Fisher Scientific). The cDNA product was added to reaction solutions containing SYBR Green mix (Thermo Fisher Scientific) and DNA primers, and then amplified using a StepOnePlus real-time PCR systems (Thermo Fisher Scientific). The DNA primer sequences were designed as shown in Supplementary Table S1. Expression of β-actin was selected as a housekeeping gene. The amounts of MDR1 and BCL2 mRNA on the siRNA@PLGA NPs-treated A2780-CP20 cancer cells were measured in a similar way as described above.

The expression levels of MDR1 and BCL2 proteins on each cancer cells were also examined by western blotting analysis. Briefly, siRNA@PLGA NPs-treated SKOV3-TR or A2780-CP20 cells were prepared in the same protocol as described above. The harvested cells were subjected to lysis by RIPA lysis buffer (Sigma) supplemented with cocktail protease inhibitors (Roche, Germany), and then the cell lysates were collected by centrifugation. Protein samples from the supernatant was resolved on SDS PAGE electrophoresis and transferred onto PVDF (polyvinyl difluoride) membranes. The membranes were immunoblotted with primary antibodies specific for P-glycoprotein or BCL2, and HRP-conjugated goat anti-rabbit IgG (Abcam) were supplemented to develop the blots. The expression levels of BCL2 or P-glycoprotein were relatively compared with those of housekeeping β-actin proteins. EZ-Capture MG (Japan) was used to analyze the western blotting images, together with Image J software.

### Cell internalization studies using confocal images and flow cytometry

For confocal microscopic images, each cell lines of SKOV3-TR and A2780-CP20 cancer cells were cultured overnight to reach 70% confluency as aforementioned. Next, the cells were transfected with the fluorescent Cy5.5-siRNA-loaded PLGA NPs (0.28 mg/mL) for 3 h. Each well was washed three times with DPBS buffer, and then the cells were fixed by PBS buffer containing 4% paraformaldehyde for 1 h. 4,6-Diamidino-2-phenylindole (DAPI) was treated to the cells for nuclear staining. Cellular internalization of the fluorescent Cy5.5-siRNA-loaded PLGA NPs was assessed using LSM710 laser-scanning confocal microscope (Carl Zeiss), which was equipped with 405 nm and 514 nm laser for DAPI and Cy5.5 respectively. For the high magnification 3D images, 63x objective and LSM710 ZEN software was used.

For flow cytometry analysis, each cell lines treated with the fluorescent Cy5.5-siRNA-loaded PLGA NPs were examined by Guava EasyCyte flow cytometer (Merck Millipore) equipped with 640 nm laser. The fluorescent Cy5.5-siRNA@PLGA NPs-treated cells were detached from plates using trypsin-EDTA, and then 1 × 10^4^ cells/set were subjected to flow cytometry analysis.

### Apoptosis assay

Induction of apoptosis was investigated on the siRNA@PLGA NPs-treated ovarian cancer cells using flow cytometry and Annexin V-FITC/PI kit (BD Pharmingen). First, MDR SKOV3-TR cancer cells were cultured overnight to reach 70% confluency as mentioned above, and then pretreated with siRNA@PLGA NPs (0.28 μg/mL) containing each 37.5 nM of MDR1 and BCL2 siRNA 24 h before paclitaxel administration (5 μM). After paclitaxel treatment, the cells were further incubated for 48 h, and then harvested. Finally, the cells stained with PI/Annexin V-FITC solution were subjected to flow cytometry analysis. The relative fractions of apoptotic and necrotic cells were shown as the mean value of three independent experiments. Similarly, MDR A2780-CP20 cancer cells were pretreated with siRNA@PLGA NPs (0.28 μg/mL) 24 h before cisplatin administration (50 μM), and then subjected to flow cytometry analysis.

### Statistical analysis

All results are represented as mean ± s.d. (n = 3) and statistical significance among groups was tested by the Student’s *t*-test. Analysis of variance, ANOVA, was carried out for multiple group comparisons. Probability values with *P* < 0.05 were considered statistically significant.

## Electronic supplementary material

Supplementary Information
