# Supplementary material for: PLGA nanoparticles co-delivering MDR1 and BCL2 siRNA for overcoming resistance of paclitaxel and cisplatin in recurrent or advanced ovarian cancer
Source: Sci Rep. 2018 May 14;8:7498. doi: 10.1038/s41598-018-25930-7 (PMC5951813; doi:10.1038/s41598-018-25930-7)
Supplement: Supplementary file 1 — Supplementary Information [file 41598_2018_25930_MOESM1_ESM.pdf]

Supplementary Information

PLGA nanoparticles co-delivering MDR1 and BCL2 siRNA for overcoming resistance of paclitaxel and cisplatin in recurrent or advanced ovarian cancer

Chitra Risnayanti[†,§,‡], Yeong-Su Jang[†,§], Jinju Lee[§] and Hyung Jun Ahn[§,*]

[§] Center for Theragnosis, Biomedical Research Institute, Korea Institute of Science and Technology, Seoul 02792, Republic of Korea

[‡] Division of Bio-Medical Science &Technology, KIST School, Korea University of Science and Technology, Seoul 02792, Republic of Korea

† Both authors contributed equally to this manuscript

* Corresponding author: Hyung Jun Ahn, PhD
Korea Institute of Science and Technology
39-1 Hawolgok-dong, Seongbuk-gu, Seoul 136-791, Republic of Korea
Tel: +82-2-958-5938; fax: +82-2-958-5909
E-mail: hjahn@kist.re.kr

anti-MDR1 siRNA duplex	5′-GGAAAAGAAACCAACUGUCdTdT-3′<br>3′-dTdTCCUUUUCUUUGGUUGACAG-5′
anti-BCL2 siRNA duplex	5′-GUACAUCCAUUAUAAGCUGUCdTdT-3′<br>3′-dTdTCAUGUAGGUAAUAUUCGACAG-5′
alternative anti-MDR1 siRNA duplex	5′-CAUGAAUCUGGAGGAAGACdTdT-3′<br>3′-dTdTGUACUUAGACCUCCUUCUG-5′
alternative anti-BCL2 siRNA duplex	5′-GGCAUAUCUAAAAAGAUAGdTdT-3′<br>3′-dTdTCCGUAUAGAUUUUUCUAUC-5′
scrambled siRNA duplex	5′-UGAAGUUGCACUUGAAGUCdTdT-3′<br>3′-dTdTACUUCAACGUGAACUUCAG-5′
MDR1 forward primer	5′-GAAATTTAGAAGATCTGATGTCAAACA-3′
MDR1 reverse primer	5′- ACTGTAATAATAGGCATACCTGGTCA -3′
BCL2 forward primer	5′-AGTACCTGAACCGGCACCT-3′
BCL2 reverse primer	5′-GCCGTACAGTTCCACAAAGG-3′
β-actin forward primer	5′-AGAGGGAAATCGTGCGTGAC-3′
β-actin reverse primer	5′-CAATAGTGATGACCTGGCCGT-3′

Table S1. Oligonucleotide sequences used in the current studies.

Table S2. IC_{50} values for resistant and sensitive ovarian cancer cell lines

Cell line	Treatment	IC_{50} (μM)
SKOV3 (sensitive)	paclitaxel alone	0.39 ± 0.0021
SKOV3-TR (resistant)	paclitaxel alone	6.98 ± 0.0294
	siRNA@PLGA NPs + paclitaxel	0.91 ± 0.0015
A2780 (sensitive)	cisplatin alone	0.082 ± 0.0047
A2780-CP20 (resistant)	cisplatin alone	55.3 ± 0.4523
	siRNA@PLGA NPs + cisplatin	12.4 ± 0.1054

Table S2. **IC_{50} values for sensitive and resistant ovarian cancer cell lines.** IC_{50} value of paclitaxel combined with PLGA nanoparticles containing MDR1 and BCL2 siRNA (siRNA@PLGA NPs) was determined on SKOV3-TR resistant cells, and compared to that on SKOV3 parent cells. siRNA@PLGA NPs were treated to the cells 24 h prior to chemotherapeutics treatment, and the cell viability of cells was measured by an MTT assay 48 h post chemotherapeutics treatment. Similarly, the IC_{50} value of cisplatin combined with siRNA@PLGA NPs was measured on A2780-CP20 resistant cells, and compared to that on A2780 parent cells. The results are shown as the mean ± s.d. (n=3).

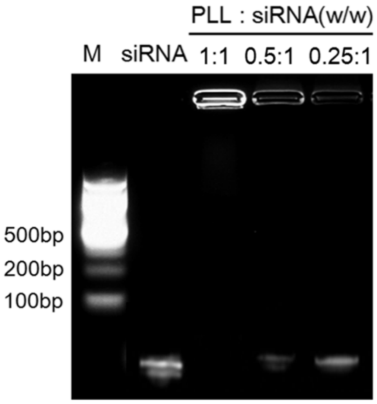

Figure S1. Agarose retardation assay showing formation of PLL/siRNA complexes. A fixed amount of siRNA was complexed with various amounts of PLL ranging from 0.25:1 to 1:1 (w/w) to determine the complexing ratio between PLL and siRNA. The siRNA/PLL complexes were subjected to 2% agarose gel electrophoresis under TBE running buffer, and then their bands were obtained by SYBR Gold staining and DNR Bio-Imaging Systems.

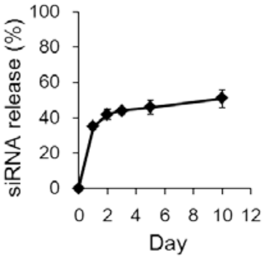

Figure S2. In vitro release profile of siRNA from siRNA@PLGA nanoparticles. A small volume of suspension of Cy5.5-siRNA@PLGA in PBS buffer was withdrawn at the indicated time points, and after the nanoparticles were centrifuged and disintegrated with DMSO, the fluorescence intensity of Cy5.5-siRNA was measured. The results are shown as the mean ± s.d. (n=3).

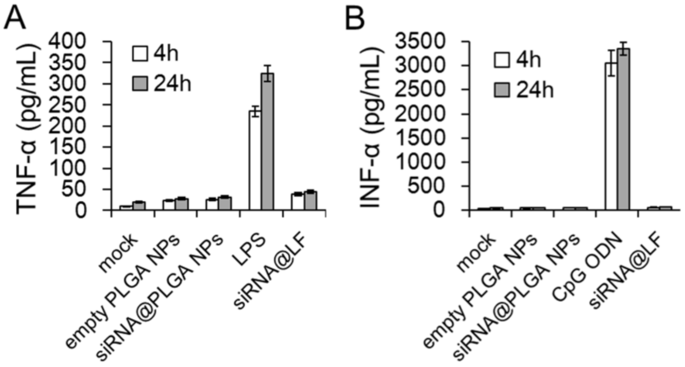

Figure S3. Proinflammatory cytokine induction studies of siRNA@PLGA nanoparticles. TNF-α or INF-α release was determined 24h or 48h after treatment with PBS (mock), empty PLGA NPs (0.28 μg/mL), siRNA@PLGA NPs (0.28 μg/mL containing each 37.5 nM of MDR1 and BCL2 siRNA), or siRNA@lipofectamine (0.2 μg/mL containing each 37.5 nM of MDR1 and BCL2 siRNA). TNF-α and INF-α induction was examined with the cell culture medium derived from mouse macrophage RAW264.7 cells and human Burkitt's lymphoma Ramos cells, respectively. As a positive control, 0.2 μg/mL lipopolysaccharide (LPS) was chosen for TNF-α, while 20 μM CpG oligodeoxynucleotides for INF-α induction. TNF-α and INF-α was quantified by TNF-α Platinum ELISA kit (eBioscience) and VeriKine human INF-α ELISA kit (Pestka Biomedical Laboratories), respectively. The results are shown as the mean ± s.d. (n=3).

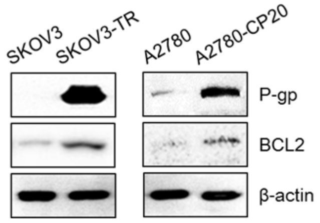

Figure S4. Intrinsic overexpression of MDR1 and BCL2 genes on SKOV3 or A2780 parent and resistant cell lines. The representative immunoblotting images show the expression of P-glycoproteins and BCL2 proteins on SKOV3 parent and resistant cells, or A2780 parent and resistant cells. In the figure are reported the cropped gels/blots obtained by each protein evaluation. All gels were run in the same experimental conditions. β-actin bands as a housekeeping gene indicate that the equal concentrations of cellular protein extracts were loaded to each lane.

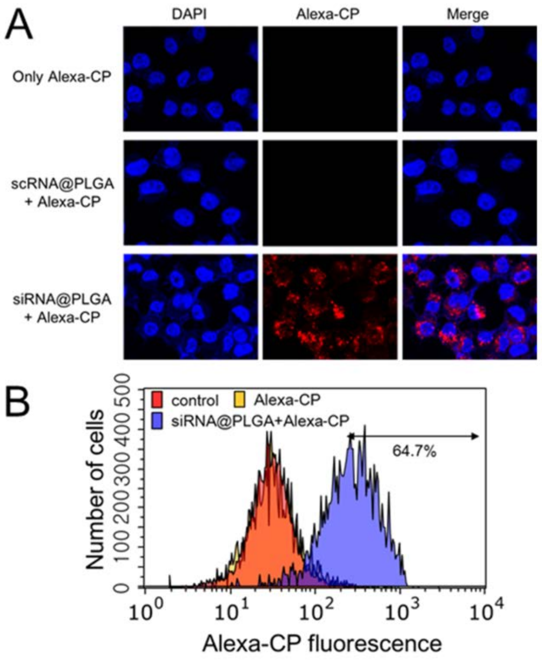

Figure S5. Increased intracellular concentration of drugs on siRNA@PLGA NPs-pretreated MDR A2780-CP20 cancer cells. (A) Confocal microscopic images demonstrating the suppression of drug efflux on MDR A2780-CP20 cells. Cells were first pretreated with siRNA@PLGA NPs, and 24 h later, administrated with 1 μM fluorescent cisplatin conjugates (Alexa Fluor 546-cisplatin, Alexa-CP) for monitoring their intracellular localization. Cellular confocal microscopic evaluation was carried out 12 h after drug treatment. As a control, suppression of drug efflux by the scrambled siRNA@PLGA NPs was also compared. (B) Flow cytometry analysis for measuring the intracellular accumulation of fluorescent cisplatin conjugates on A2780-CP20 cells. A prefixed gate region was allowed only for Alexa Fluor 546 fluorescence. A representative histogram among three independent experiments was shown here.

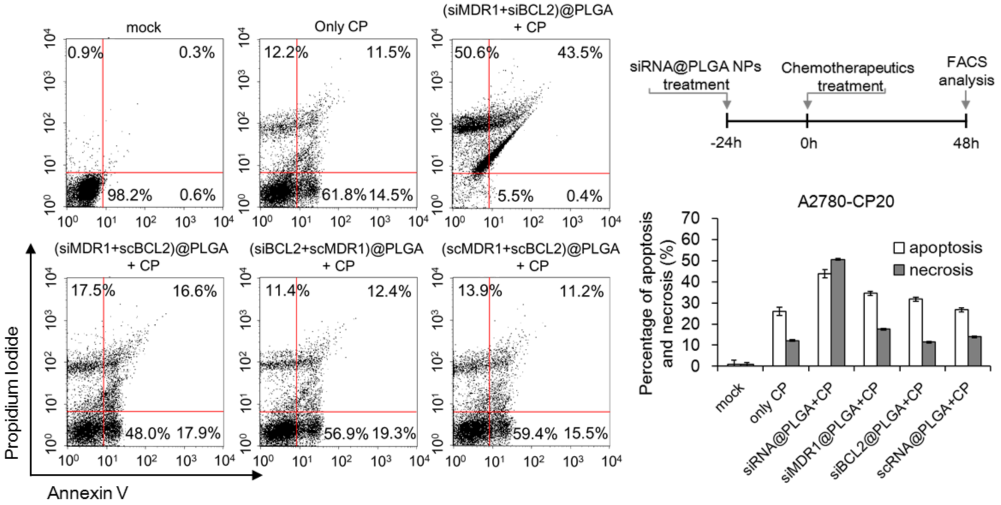

Figure S6. Induction of apoptosis on MDR A2780-CP20 cells by combination treatment. MDR A2780-CP20 cells were sequentially treated with PLGA NPs containing MDR1 and BCL2 siRNA (37.5 nM) and cisplatin (50 μM), as shown in a schematic diagram. Flow cytometry analysis reveals the percentage of fully apoptotic, early apoptotic, and necrotic cells, which are represented in the corresponding quadrant. A representative data set is shown here among three independent experiments. The results are shown as the mean ± s.d. (n=3). The percentage of apoptosis in the plot includes of fully and early apoptotic cells.

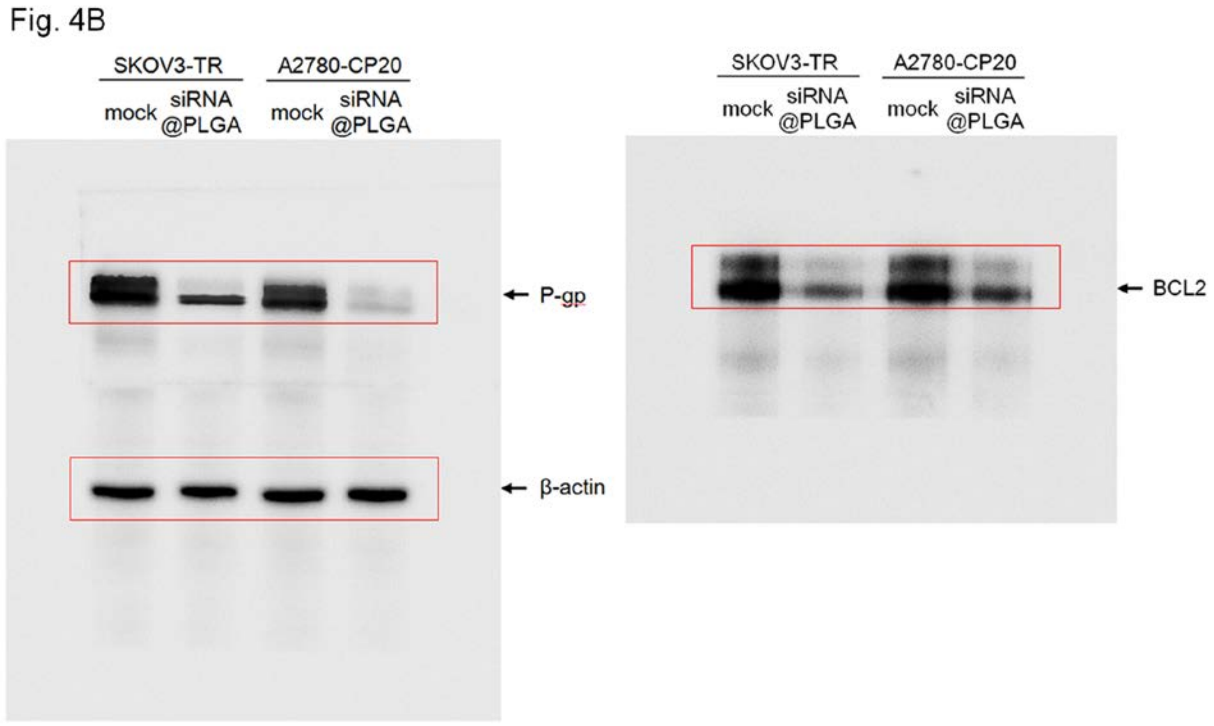

Figure S7. The expression of P-glycoproteins and BCL2 proteins on SKOV3-TR and A2780-CP20 cells 24 h post-transfection. The representative full blot images of Figure 4B are shown here.
